# The proliferative effect of cortisol on bovine endometrial epithelial cells

**DOI:** 10.1186/s12958-019-0544-1

**Published:** 2019-11-22

**Authors:** Junsheng Dong, Jun Li, Jianji Li, Luying Cui, Xia Meng, Yang Qu, Heng Wang

**Affiliations:** 1 College of Veterinary Medicine, Yangzhou University, Yangzhou, 225009 Jiangsu China; 2 Jiangsu Co-innovation Center for the Prevention and Control of Important Animal Infectious Diseases and Zoonoses, Yangzhou, 225009 Jiangsu China

**Keywords:** Bovine endometrial epithelial cell, Cortisol, Growth factors, Wnt/β-catenin, PI3K/AKT, Proliferation

## Abstract

**Background:**

Bovine endometrial epithelial cells (BEECs) undergo regular regeneration after calving. Elevated cortisol concentrations have been reported in postpartum cattle due to various stresses. However, the effects of the physiological level of cortisol on proliferation in BEECs have not been reported. The aim of this study was to investigate whether cortisol can influence the proliferation properties of BEECs and to clarify the possible underlying mechanism.

**Methods:**

BEECs were treated with different concentrations of cortisol (5, 15 and 30 ng/mL). The mRNA expression of various growth factors was detected by quantitative reverse transcription-polymerase chain reaction (qPCR), progression of the cell cycle in BEECs was measured using flow cytometric analysis, and the activation of the Wnt/β-catenin and phosphatidylinositol 3-kinase (PI3K)/protein kinase B (AKT) signaling pathways was detected with Western blot and immunofluorescence.

**Results:**

Cortisol treatment resulted in upregulated mRNA levels of vascular endothelial growth factor (VEGF) and connective tissue growth factor (CTGF); however, it had no influence on transforming growth factor-beta1 (TGF-β1). Cortisol (15 ng/mL) accelerated the cell cycle transition from the G0/G1 to the S phase. Cortisol upregulated the expression of β-catenin, c-Myc, and cyclinD1 and promoted the phosphorylation of PI3K and AKT.

**Conclusions:**

These results demonstrated that cortisol may promote proliferation in BEECs by increasing the expression of some growth factors and activating the Wnt/β-catenin and PI3K/AKT signaling pathways.

## Background

The mammalian uterus exhibits a unique regenerative ability as it undergoes the cyclic program of degeneration and regeneration. During the period of parturition, bovine endometrial epithelial cells (BEECs) are partially destroyed [1]. Subsequently, the damaged endometrium is effectively repaired without remaining scar tissue or loss of function [2]. This repair is essential to prepare for another pregnancy and to form natural defense barriers against various pathogenic microorganisms.

Cortisol acts as an endogenous glucocorticoid, which can be raised in the organism by stress [3]. The blood levels of cortisol increase in parturients. A previous study showed that glucocorticoids inhibited cell proliferation due to their cytotoxic effects and their induction of cell-cycle arrest and apoptosis [4]. However, an increasing number of studies have shown that glucocorticoids can promote proliferation in a variety of cell types [5–7]. It has been demonstrated by Petersen et al. that low dose dexamethasone treatment led to a moderate increase in the proliferation of cultured human lens epithelial cells [7]. Komiyama et al. reported that cortisol suppressed apoptosis of luteal cells to maintain bovine corpus luteum function at the early and midluteal stage [8]. These studies explain why a low concentration of cortisol is added into some culture media as a growth enhancer [9]. The effect of glucocorticoids depends on the differentiation status of the cells [10]. Glucocorticoid treatment reduced undifferentiated cell proliferation, while it promoted differentiation cell survival [11]. Ciliberti et al. proved that a physiological cortisol concentration can promote peripheral blood mononuclear cell proliferation after stress [12]. Many other studies have reported that cortisol can regulate female reproductive functions in cattle [13–15]. Lee et al. showed that cortisol may act as a luteoprotective factor because it can inhibit basal and TNFα-induced PGF2α production in bovine endometrial stromal cells [16]. Duong et al. found that the function of the bovine corpus luteum was positively influenced by cortisol, which led to higher rates of embryo implantation and higher rates of pregnancy in heifers [17]. However, fewer studies have shown the effect of cortisol on the proliferation of BEECs.

After shedding of the allantochorion, the slough of the necrotic superficial endometrial drives the loss of the endometrial surface epithelial covering, so the growth of BEECs is required for the repair process. New blood vessel formation is typical for the endometrium, and the blood vessels supply oxygen to new tissue and transport immune cells to inhibit infection and inflammation [18]. VEGF is a specific mitogen of endothelial cells that plays an important role in normal and pathological angiogenesis [19]. It can also regulate normal endometrial angiogenesis. CTGF is a multifunctional growth factor that is expressed in a variety of cells and tissues, such as epithelial and secretory cells, the liver parenchyma, and vascular cells. During wound repair, CTGF expression is obviously elevated to promote wound healing, connective tissue cell proliferation and cell adhesion [20, 21]. The TGF-βs can regulate proliferation and differentiation in a variety of cell types [22]. TGF-β1 has an important function in endometrium growth. It has been reported that TGF-β1 functions as a strong upstream inducer of CTGF [23, 24].

Wnt signaling is connected with repair processes in many organ systems [25]. In primate and mice, it has been demonstrated that the Wnt/β-catenin signaling pathway is involved in the process of endometrial repair, which shows dynamic changes in the endometrium during the regeneration of endometrial epithelium [26, 27]. In the resting state, β-catenin is localized in the cytoplasm, where it combines with a destruction complex (Axin, adenomatosis polyposis coli, glycogen synthase kinase 3β and casein kinase 1α). Once the Wnt/β-catenin signaling pathway is activated, the resultant signal is transduced to the destruction complex to prevent β-catenin phosphorylation and degradation [28]. Then, free cytosolic β-catenin enters the nucleus to bind the T-cell factor/lymphoid enhancer factor (TCF/LEF) family and regulate the expression of downstream target genes, such as c-Myc and cyclinD1, which are closely involved in proliferation and the cell cycle [29, 30]. Accumulating evidence has confirmed that the PI3K/AKT signaling pathway is an important intracellular signaling pathway in the regulation of numerous cellular functions, including proliferation, adhesion, migration, invasion, metabolism and survival [31–33]**.** PI3K is the main upstream molecule that activates AKT, and then AKT induces cell growth and survival.

The aim of this study was to investigate the proliferative effect of cortisol on BEECs and to clarify the possible mechanisms of the effects. Our study was designed to detect changes in the mRNA levels of growth factors (VEGF, CTGF and TGF-β1), the cell cycle, and the critical proteins of the Wnt/β-catenin and PI3K/AKT signaling pathways after treatment with different concentrations of cortisol, and we assessed whether cortisol could promote BEEC proliferation in vitro.

## Methods

### Isolation and culture of endometrial epithelial cells

Bovine uteri with no gross evidence of genital disease or microbial infection were collected from an abattoir and kept on ice until further processing at the laboratory. Postpartum uteri were discarded due to contamination of the uterus, damage to the endometrium and local inflammation. The uterus was collected at days 1–4 of the estrous cycle (day 1 represents the ovulation day), with ovarian stage I used for cell culture because at that point [34], peripheral plasma progesterone concentrations are similar to those of a postpartum bovine [1]. In brief, the uterine horn was cut into 3–4 cm long sections. Tissues were digested with 0.1% protease from *Streptomyces griseus* (P5147, Sigma, USA), 200 units/mL penicillin and 200 μg/mL streptomycin dissolved in DMEM-F12 (D8900, Sigma, USA). After an 18-h incubation at 4 °C, the uterine horn was incised longitudinally to expose the epithelium. The endometrium was scraped gently using surgical blade and ophthalmic tweezers. Harvested endometria were centrifuged at 100×g for 5 min and then washed twice with PBS. Then, the cell pellet was collected. Cells were seeded into 25 cm^2^ flasks in Dulbecco’s modified Eagle’s medium/nutrient mixture F-12 containing 15% fetal bovine serum (FBS, Gibco, USA), 50 U/mL penicillin/streptomycin and cultured at 37 °C with 5% CO_2_. The medium was changed every 1–2 days until the cells reached approximately 90% confluence. The purification of BEECs was determined by detecting CK-18 using immunohistochemistry, and the proportion of epithelial cells was determined to be greater than 99%. The BEECs were seeded and treated until they reached 80% confluence. The BEECs were isolated and cultivated independently. Each set of cultured cells was from a single uterus and represented a uterus in the experiment. The cells of each independent experiment were from a single uterus.

### RNA extraction and quantitative PCR (qPCR)

Our previous study verified that 5 ng/mL (basal physiological level), 15 ng/mL (physiological level at parturition), and 30 ng/mL (supra-physiological levels, such as at exogenous administration or pathological condition) concentrations of cortisol have no cytotoxic effects on BEECs [35]. The BEECs were treated with cortisol (5, 15 and 30 ng/mL) for 0, 3, 12, and 18 h. After incubation with cortisol (H0888, Sigma, USA), total RNA was extracted according to the manufacturer’s instructions using TRIzol reagent (ET111, TRAN, China). The quantity and purity analysis of the extracted RNA were checked using a Nanodrop 2000 spectrophotometer (Thermo, USA). The ratio of absorption (A260/A280) was determined to be between 1.8 and 2.1, and then the RNA (900 ng) was converted to cDNA as previously described [35]. The cycling conditions were as follows: 95 °C for 30 s, 40 cycles of 95 °C for 5 s, 60 °C for 30 s. The reaction system included 12.5 μL of SYBR Green PCR mix, 1 μL of each primer, and 1 μL of cDNA template in a final volume of 25 μL per reaction (RR820A, Takara, Japan). The 2^-△△Ct^ method was used to analyze the relative gene expression (target gene expression normalized to the expression of the endogenous control gene) [36]. The qPCR experiments were performed in triplicate. The sequences of the primers are presented in Table 1.

**Table 1:** The list of primer sequences used for amplification of qPCR

Gene	Forward primers	Reverse primer	Accession number	Product size (bp)
β-actin	CATCACCATCGGCAATGAGC	AGCACCGTGTTGGCGTAGAG	NM_173979.3	156
VEGF	CCTGATGCGGTGCGGGGGCT	TGGTGGTGGCGGCGGCTATG	NM_001316992.1	372
CTGF	AGCTGACCTGGAGGAGAACA	GTCTGTGCACACTCCGCAGA	NM_174030.2	139
TGF-β1	CGAGCCCTGGACACCAACTA	AGGCAGAAATTGGCGTGGTA	NM_001166068.1	137

### Cell cycling analysis

The BEECs were treated with cortisol (5, 15 and 30 ng/mL) for 24 h. Then, the cells were collected, washed twice with cold PBS, and fixed in 70% ethanol at 4 °C for 24 h. Then, the cells were washed twice with cold PBS and incubated with RNaseA and propidium iodide (C1052, Beyotime, China) for 30 min in the dark at 37 °C. The stage of the cell cycle was determined by flow cytometry (LSRFortessa, BD Biosciences, USA).

### Western blot analysis

The BEECs were treated with cortisol as described above, and the total proteins were extracted and quantified using a BCA protein assay kit (P0010, Beyotime, China). Proteins (20–30 μg) were separated by 10% SDS-polyacrylamide gels and transferred to polyvinylidene difluoride (PVDF) membranes (Millipore, Germany). The membranes were incubated in 5% nonfat milk diluted with TBST (0.1% Tween-20 in Tris-buffered saline) to block nonspecific binding. The membranes were incubated with primary antibodies specific for β-catenin (1:5000 dilution in 5% BSA), p-AKT (1:2000 dilution in 5% BSA), c-Myc, cyclinD1, p-PI3K, PI3K, AKT and β-actin (all at 1:1000 dilution in 5% BSA) at 4 °C overnight, and then they were incubated with HRP-conjugated secondary antibodies (all at 1:2000 dilution in 5% nonfat milk) at room temperature for 1 h. The following antibodies were used: β-catenin (ab32572; Abcam; U.K.), c-Myc, cyclinD1, p-PI3K, PI3K, p-AKT, AKT and β-actin (#5605, #2978, #4228, #4292, #4060, #4691, #4970, respectively; Cell Signaling Technology, USA).

### Immunofluorescence staining

The BEECs grew on cover slips in 24-well cell culture plates. Cells were treated with cortisol at a concentration of 15 ng/mL for 30 min. After treatment, cells were fixed with 4% paraformaldehyde for 30 min. After washing with PBS, cellular membranes were permeabilized with 0.1% Triton X-100 for 10 min, and cells were blocked with 5% bovine serum albumin for 30 min at room temperature. After that, cells were incubated with anti-β-catenin (all at 1:250 in blocking solution) at 4 °C overnight. After washing with PBS three times, cells were incubated with a FITC-conjugated secondary antibody (A0423, Beyotime, China) for 1 h at room temperature. The cell nuclei were stained with DAPI (C1005, Beyotime, China). The cells were analyzed with a fluorescence microscope (Leica TCS SP8; Leica Corporation, Germany).

### Statistical analysis

Uteri were sampled from at least 3 cows. Three replicates (different sets of culture cells) were used for analysis, and the same set of culture cells were repeated 3 times within each group. All data were analyzed as the mean ± standard error of the mean (SEM). The groups were compared by one-way ANOVA, which was followed by Dunnett’s test (SPSS 17.0 software). A *p*-value of less than 0.05 was considered statistically significant.

## Results

### mRNA expression of VEGF, CTGF and TGF-β1 in BEECs is induced by cortisol

**Fig. 1:**
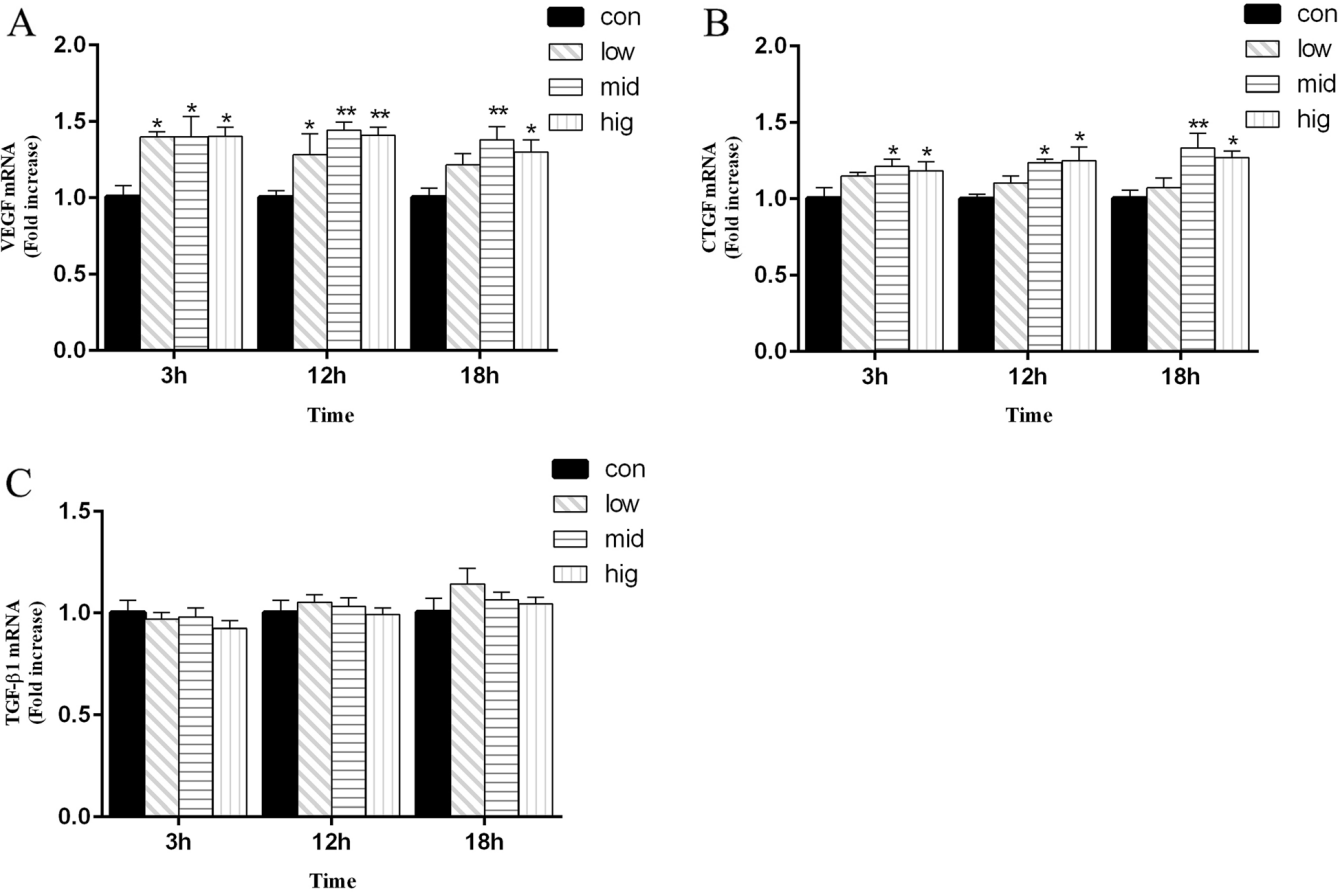
Effects of cortisol on the mRNA expression of VEGF (**a**), CTGF (**b**) and TGF-β1(**c**) in bovine endometrial epithelial cells. The bovine endometrial epithelial cells were treated with cortisol (5, 15 and 30 ng/mL) for 0, 3, 12, or 18 h. RNA was extracted and analyzed by qPCR. con = control cells without any processing; low = 5 ng/mL cortisol; mid = 15 ng/mL cortisol; high = 30 ng/mL cortisol. Three uteri (different sets of culture cells) were used for analysis. The data are presented as the means ± SEM. * *p* < 0.05, ** *p* < 0.01 vs the control group

To investigate the potential impact of cortisol on BEEC proliferation, we examined the mRNA levels of VEGF, CTGF and TGF-β1 by qPCR. As shown in Fig. 1, at 3 h and 12 h, the mRNA levels of VEGF were increased (*p* < 0.05) after 5 ng/mL, 15 ng/mL and 30 ng/mL cortisol treatment compared to those in the control group. At 18 h, VEGF expression was higher (*p* < 0.05) than it was in the control group after 15 ng/mL and 30 ng/mL but not after 5 ng/mL cortisol treatment. At 3 h, 12 h and 18 h, the mRNA levels of CTGF were significantly upregulated (*p* < 0.05) after 15 ng/mL and 30 ng/mL cortisol treatment. The mRNA levels of TGF-β1 in the experimental groups were no different than they were in the control group at the indicated time points.

### Effect of cortisol on the cell cycle in BEECs

**Fig. 2:**
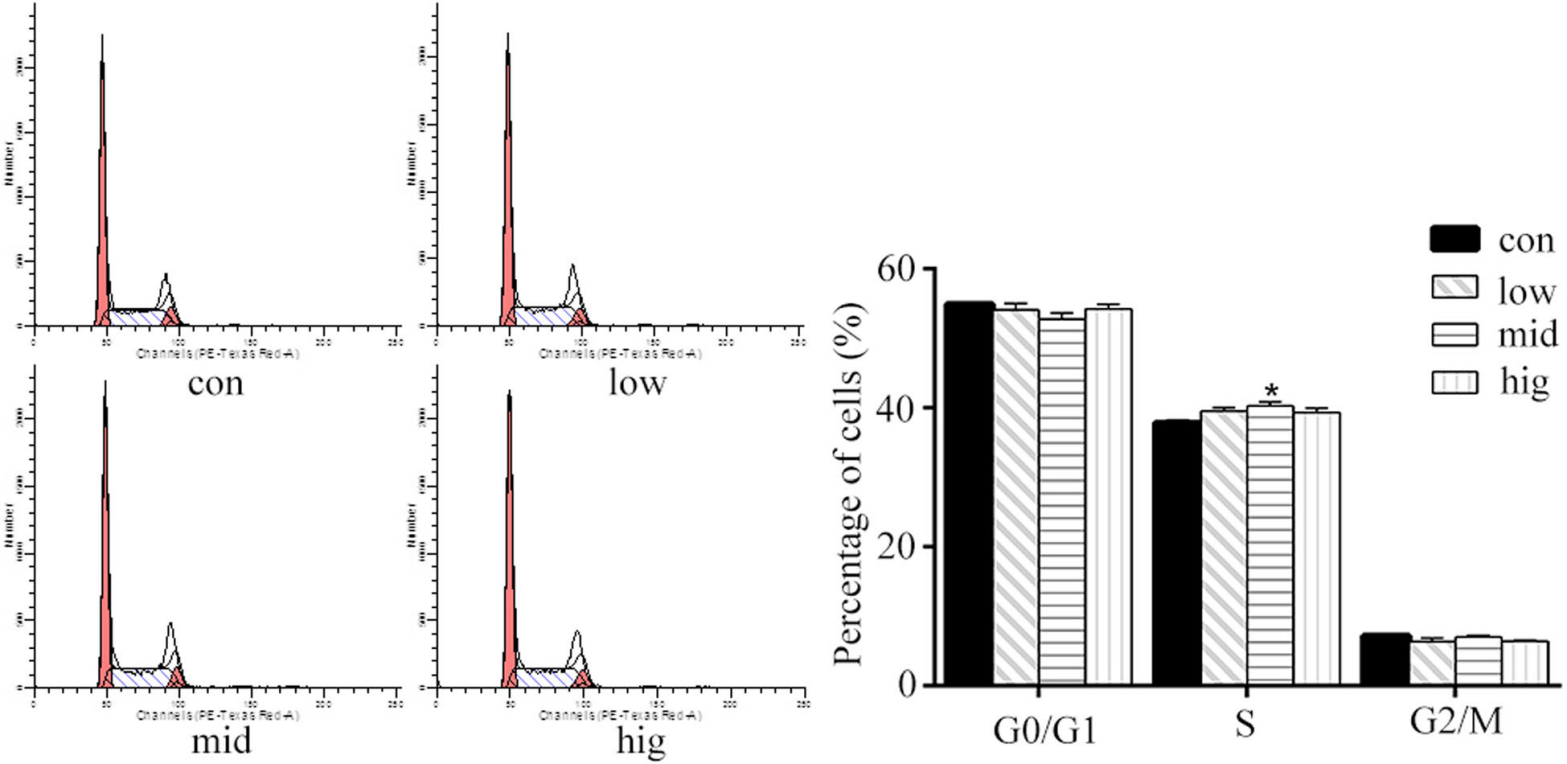
Effects of cortisol on cell cycle distribution in bovine endometrial epithelial cells. The bovine endometrial epithelial cells were treated with cortisol (5, 15 and 30 ng/mL) for 24 h. The cell cycle distribution was examined by flow cytometry. Con = control cells without any processing; low = 5 ng/mL cortisol; mid = 15 ng/mL cortisol; high = 30 ng/mL cortisol. Three uteri (different sets of culture cells) were used for analysis. The data are presented as the means ± SEM. * *p* < 0.05 vs the control group

To explore the possible roles of cortisol in controlling BEEC proliferation, we measured the cell cycle distribution by flow cytometry (Fig. 2). The results demonstrated that 15 ng/mL cortisol significantly increased (*p* < 0.05) the proportion of cells in S phase, and 5 ng/mL and 30 ng/mL cortisol groups also showed a similar tendency. These data indicated that 15 ng/mL cortisol might promote BEEC growth by accelerating the G0/G1 to S phase transition in the cell cycle.

### Cortisol activates the Wnt/β-catenin signaling pathway in BEECs

**Fig. 3:**
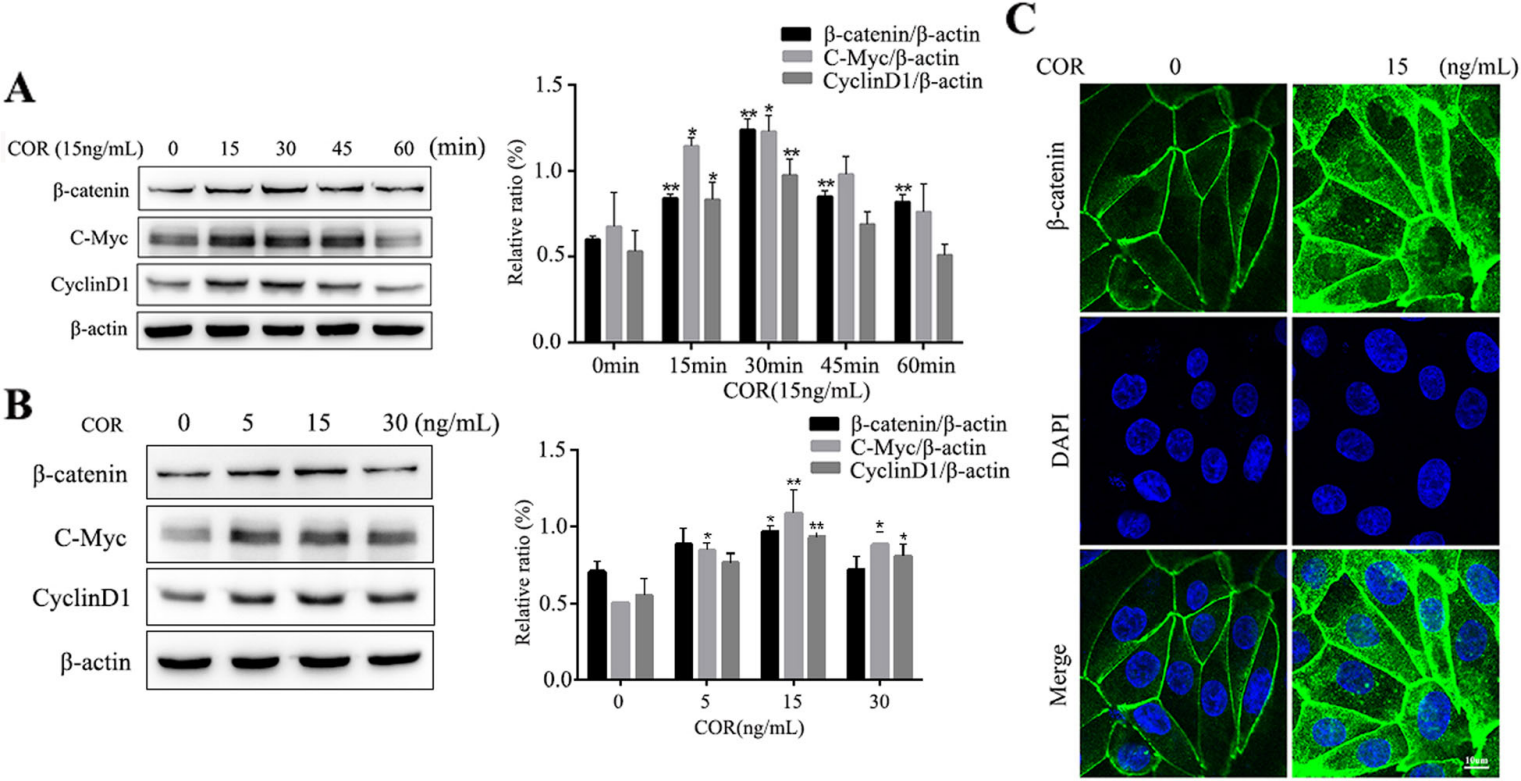
The effect of cortisol on the activity of the Wnt/β-catenin pathway in bovine endometrial epithelial cells. (**a**) Cells were treated with cortisol (15 ng/mL) for 0, 15, 30, 45 and 60 min. (**b**) Cells were treated with cortisol (5, 15 and 30 ng/mL) for 30 min. The β-catenin, c-Myc and cyclinD1 levels were determined by Western blotting analysis. β-actin was used as the internal control. (**c**) Cells were treated with cortisol (15 ng/mL) for 30 min. The β-catenin levels were evaluated by confocal microscopy. Three uteri (different sets of culture cells) were used for analysis. The data are presented as the means ± SEM. * *p* < 0.05, ** *p* < 0.01 vs the control group

To determine whether the Wnt/β-catenin signaling pathway was involved in regulating proliferation in BEECs, the key proteins in the signaling pathway were detected using Western blot analysis. The results in Fig. 3a showed that the protein levels of β-catenin at all time points significantly increased (*p* < 0.01) with 15 ng/mL cortisol treatment, and the expression levels of c-Myc and cyclinD1 also increased (*p* < 0.05) at the 15 min and 30 min time points. The levels of β-catenin, c-Myc and cyclinD1 proteins reached their peak at the 30 min time point. As shown in Fig. 3b, the β-catenin protein levels were increased (*p* < 0.05) in the 15 ng/mL cortisol treatment group compared with the control group. The expression of c-Myc was increased (*p* < 0.05) following cortisol treatment at 5 ng/mL, 15 ng/mL, and 30 ng/mL compared to the control groups. Meanwhile, the expression of cyclinD1 was increased (*p* < 0.05) following cortisol treatment at 15 ng/mL and 30 ng/mL compared to the control groups. The level of β-catenin in the cell nucleus and cytoplasm was higher in the treated group than in the control group (Fig. 3c).

### Cortisol activates the PI3K/AKT signaling pathway in BEECs

To study the potential mechanism underlying the proliferative effect of cortisol on BEECs, the activation of the PI3K/AKT signaling pathway was examined by Western blot analysis.

**Fig. 4:**
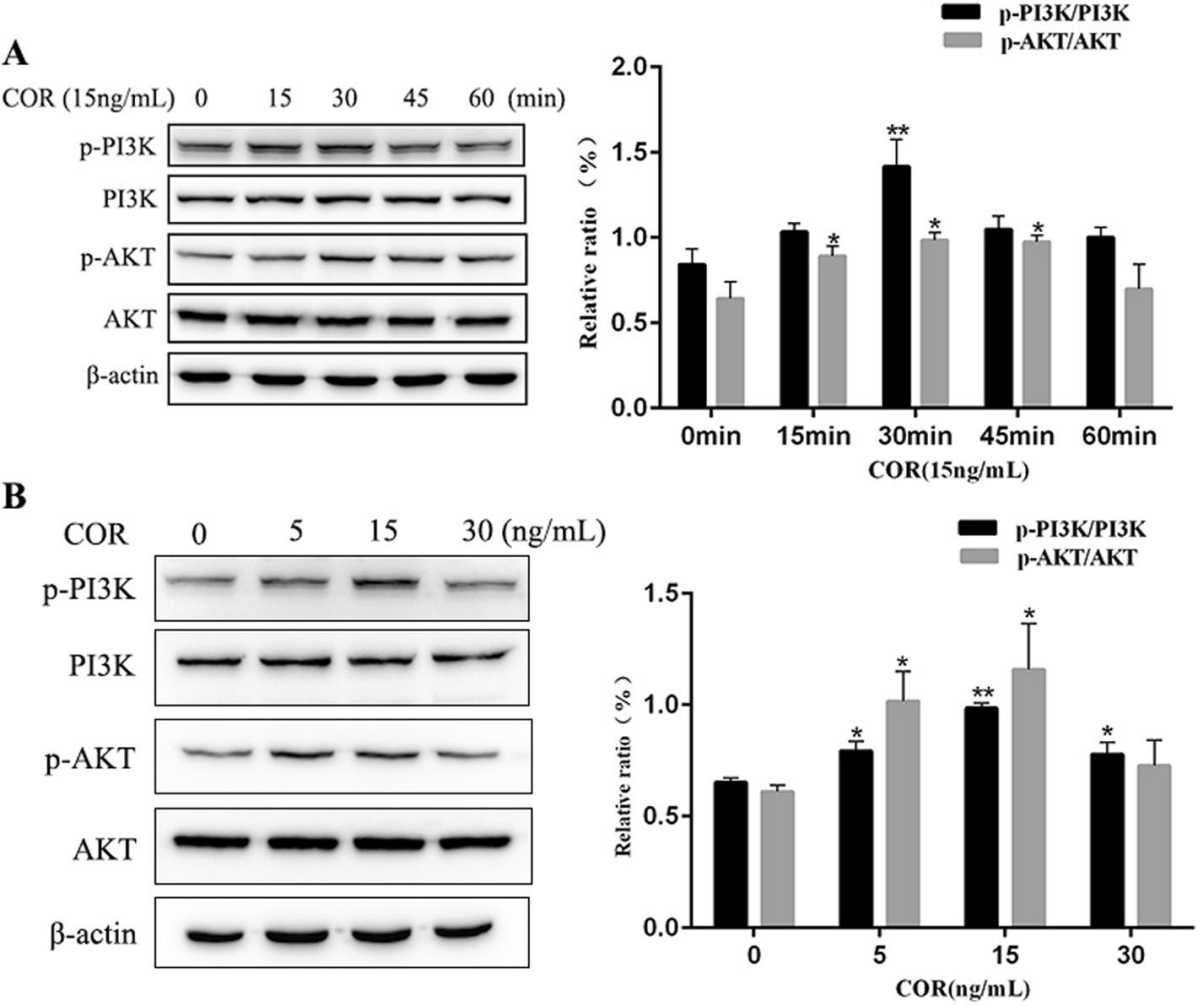
The effect of cortisol on PI3K and AKT phosphorylation in bovine endometrial epithelial cells. (**a**) Cells were treated with cortisol (15 ng/mL) for 0, 15, 30, 45 and 60 min. (**b**) Cells were treated with cortisol (5, 15 and 30 ng/mL) for 30 min. The p-PI3K, PI3K, p-AKT and AKT levels were determined by Western blotting analysis. The total PI3K or AKT protein levels were used as the internal control. Three uteri (different sets of culture cells) were used for analysis. The data are presented as the means ± SEM. * *p* < 0.05, ** *p* < 0.01 vs the control group

As shown in Fig. 4a, the phosphorylation level of PI3K was elevated (*p* < 0.01) after the 30 min cortisol treatment. Compared to that in the control groups, the phosphorylation level of AKT was elevated (*p* < 0.05) after the cortisol treatment at 15, 30 and 45 min. The phosphorylation levels of PI3K and AKT reached a peak with the 15 ng/mL cortisol treatment at the 30 min time point. The results in Fig. 4b showed that following the incubation with different concentrations (5 ng/mL, 15 ng/mL, and 30 ng/mL) of cortisol, the phosphorylation level of PI3K was significantly increased (*p* < 0.05) compared with that in the control group. Compared with that in the control group, the phosphorylation level of AKT was elevated (*p* < 0.05) following 5 ng/mL and 15 ng/mL treatments.

## Discussion

Cortisol is involved in various complex biological effects in mammals, such as growth, immune response, and metabolism. In this study, we demonstrated that cortisol can promote VEGF and CTGF gene expression and active Wnt/β-catenin and PI3K/AKT signaling pathways, which can promote cell proliferation.

Growth factors (VEGF, CTGF and TGF-β1) exert some regulatory roles in proliferation, differentiation, matrix repair and remodeling [20, 37, 38]. Our study showed that cortisol can upregulate the mRNA levels of VEGF and CTGF, but the mRNA levels of TGF-β1 were not significantly upregulated. Although it was reported that cortisol suppressed angiogenesis by increasing the levels of anti-angiogenic genes [39], this particular effect might be related to the cell-specific manner and dosage of cortisol. Bernabé et al. reported that pharmacological doses of cortisol reduced VEGF production, while cortisol could induce a significant increase of VEGF when administered at the concentration observed during physiological stress [40]. A similar effect has been reported by Fehrholz et al., in which glucocorticoids were observed to obviously increase CTGF mRNA levels in lung epithelial cells, but no effect was detected on TGF-β1 mRNA expression [41]. Dammeier et al. found that glucocorticoids induced CTGF mRNA expression independent of TGF-β1 [24]. It has been reported that steroid hormones regulate endometrial recovery, that growth factors (VEGF, CTGF and TGF-β1) are necessary for tissue formation and angiogenesis [18], and that the expression levels of these growth factors were increased in the activated repair state of BEECs [42]. Thus, cortisol could increase VEGF and CTGF mRNA levels to promote BEEC proliferation and growth in vitro.

It is widely accepted that the Wnt/β-catenin signaling pathway plays an obvious role in the proliferative phase of wound healing [43]. Chen et al. suggested that Wnts are vital factors in the development of the uterus and in embryo implantation [44]. It has been shown that cyclinD1 and c-Myc are required for the transition of G1/S and G2/M phases, respectively [45, 46]. In the present study, we found that the proportion of BEECs in S phase increased after cortisol treatment. It is a common phenotype in cancer cells that facilitating G1/S phase transition can promote cancer cell proliferation [47]. These results suggested that cortisol promoted BEEC proliferation. The data showed that compared to control cells, the levels of β-catenin, c-Myc and cyclinD1 were significantly increased after 15 ng/mL cortisol stimulation with a peak at 30 min of treatment, which indicated an obviously enhanced activation of Wnt/β-catenin that led to high expression of downstream proteins. Cortisol increased the protein levels of β-catenin, c-Myc and cyclinD1 at 30 min of treatment, which may be concentration-related. Wnt/β-catenin pathway activation had the most dramatic effect with the 15 ng/mL cortisol treatment. However, whether different concentrations of cortisol could induce different effects requires further investigation. In addition, the β-catenin protein levels were obviously elevated in the nucleus and cytoplasm after the 15 ng/mL cortisol treatment, which further demonstrated activation of the Wnt/β-catenin signaling pathway. These results were consistent with previous studies that showed accumulated β-catenin in the cytoplasm subsequently translocated to the nucleus to activate its target genes [43, 48]. Taken together, the present study showed that cortisol could regulate the Wnt/β-catenin signaling pathway to increase BEEC proliferation.

A previous study demonstrated that cell proliferation is regulated by a reduction in apoptosis during early wound healing [49]. The PI3K/AKT pathway is an important regulator of cell proliferation, apoptosis and cell cycle [50, 51]. Evidence has shown that the pathway is closely related to proliferative diseases, such as cancer [52, 53]. Our results indicated that the phosphorylation levels of PI3K and AKT peaked at 30 min with the 15 ng/mL cortisol treatment. Furthermore, various concentrations of cortisol increased the phosphorylation levels of PI3K and AKT at the indicated time points, with a peak at 15 ng/mL. Similar to a previous report, glucocorticoids can activate the PI3K/AKT pathway to protect against apoptosis [54]. These findings suggested that activation of PI3K/AKT was enhanced and that it participated in multiple downstream pathways in BEECs induced by cortisol. However, further investigation should be performed.

## Conclusions

The present study demonstrated the proliferative effect of cortisol on bovine endometrial epithelial cells. This effect may be achieved by increasing the expression of growth factors (VEGF and CTGF) and activating the Wnt/β-catenin and PI3K/AKT signaling pathways.

## Data Availability

The datasets used and analyzed during the current study are available from the corresponding author on reasonable request.

## Abbreviations

AKT: Protein kinase B
BEECs: Bovine endometrial epithelial cells
BSA: Bovine serum albumin
CTGF: Connective tissue growth factor
DMEM-F12: Dulbecco’s modified Eagle’s medium and Ham’s F-12 nutrient mixture
FBS: Fetal bovine serum
PBS: Phosphate-buffered saline
PI3K: Phosphatidylinositol 3-kinase
PVDF: Polyvinylidene difluoride
qPCR: Quantitative reverse transcription-polymerase chain reaction
TGF-β1: Transforming growth factor-beta1
VEGF: Vascular endothelial growth factor
